# Mindful self-care among oncology nurses in China: a latent profile analysis

**DOI:** 10.1186/s12912-024-02156-9

**Published:** 2024-07-14

**Authors:** Yan Shi, Peng Wang, Lamei Liu, Mengmeng Li

**Affiliations:** 1 School of Nursing and Health, Zhengzhou University, No. 101 Science Avenue, Zhengzhou, Henan Province China; 2 School of Nursing, Xinxiang Medical University, Xinxiang, China; 3 Zhengzhou Central Hospital Affiliated to Zhengzhou University, Zhengzhou, China

**Keywords:** Mindful self-care, Chinese, Oncology nurses, Latent profile analysis, Influencing factors

## Abstract

**Background:**

Oncology nurses are considered the group with the highest risk for moral distress, compassion fatigue and burnout. Mindful self-care may help oncology nurses improve their well-being and solve psychological problems. However, the investigation and in-depth analysis of mindful self-care among oncology nurses in China is lacking.

**Objectives:**

To identify heterogeneity groups of oncology nurses on mindful self-care ability and examine the sociodemographic correlation to these profiles.

**Design:**

Cross-sectional descriptive study.

**Participants:**

The study was carried out among oncology nurses in two affiliated comprehensive hospitals and one affiliated oncology hospital. A total of 839 oncology nurses were enrolled in this survey.

**Methods:**

From January to May 2023, a cross-sectional study was carried out among oncology nurses using convenient sampling. The subjects were given the brief Mindful Self-Care Scale (B-MSCS) and the General Demographic Information Questionnaire. Latent profile analysis using the Mplus 7.4 program was used to separate oncology nurses’ mindful self-care into a variety of subgroups. The SPSS 25.0 statistical program was used to analyze the data. One-way ANOVA and the chi-square test were performed to compare the score of B-MSCS in each class and the difference in sociodemographic characteristics among the subgroups. Multinomial logistic regression was used to examine the influence of the sociodemographic variables on each class.

**Results:**

The total score of the B-MSCS was 76.40 ± 13.19. The support structure dimension had the highest score, with an average mean value of 3.60, and physical care had the lowest score at 2.57. The findings of the latent profile analysis showed that respondents were divided into three classes, moderate mindful self-care(51.2%), low-low mindful relaxation(14.8%), and high-high mindfulness self-awareness(34.0%). Across scale scores and dimensions, three groups demonstrated statistically significant differences (*p* < 0.05). Univariate analysis revealed significant differences between the three profiles in terms of professional title, position, concern about self-care, interest in mindfulness, and experience with meditation (*p* < 0.05). Profile membership was predicted by 3 factors, namely, self-care status, interest in mindfulness, and experience with meditation.

**Conclusion:**

The mindful self-care among oncology nurses can be categorized into three latent profiles: moderate mindful self-care, low-low mindful relaxation, and high-high mindfulness self-awareness. Multinomial logistic regression results indicated that whether oncology nurses concern about self-care, interest in mindfulness and have experience with meditation influenced different latent profiles. Nursing manager should develop targeted intervention based on the typological characteristics of the oncology nurses to improve their mindful self-care ability and mental health.

**Supplementary Information:**

The online version contains supplementary material available at 10.1186/s12912-024-02156-9.

## Introduction

As the International Agency for Research on Cancer (IARC) released, over 35 million new cancer cases were predicted in 2050 [1]. The burden of cancer in China presents a coexistence of developed and developing countries. The situation of cancer prevention and control is still serious in China [2]. The growing cancer burden will highlight the shortage on oncology healthcare professionals and bring more stress factors to oncology nurses. Researches have shown that oncology nurses always have no time to rest, experience stress [3] and low levels of personal accomplishment in this busy work environment [4]. They often face patient deaths and must listen to sad or despairing stories. So they are always expected to be intensely empathic, and should display or suppress their emotions intermittently [5]. Research also suggested that terminal care, death anxiety, a lack of social support, and ethical issues related to patient care were all work-related stressors that oncology nurses face [6–11]. These stressors can affect nurses’ psychological status and job satisfaction [12], and cause irritability, sleeplessness and fatigue [13] which will influence oncology nurses’ caring behaviors [14]. Therefore, oncology nurses were considered the group with the highest risk for moral distress [5], compassion fatigue(CF) and burnout [6, 7]. They must know how to mentally remove themselves from the tragic moments and emotional devastation surrounding them [5]. And they should develop the experience, skills, social support and control [11] needed to manage their psychological health and work-related stress [15]. To further develop holistic nursing care, enhancing oncology nurses’ healthy coping strategies at both the individual and organizational level are very important [16].

Self-care activities and practicing mindfulness may be an effective way to reduce the occupational burnout [17, 18] which can help nursing professionals to maintain good boundaries and be fully present during care [19]. Practicing self-care is also a requisite for nurses as they face the extreme physical, mental, and emotional challenges [20]. Research on hospice care professionals has shown that those who frequently use self-care strategies experience a higher professional quality of life [21]. Self-Care Intervention can assist oncology nurses in the provision of compassionate caring for their patients, potentially minimize compassion fatigue [22], enhance their level of self-compassion, and improve their mental health [23]. Mindfulness is also a form of self-care [19] which can also be used as a defensive style by oncology professionals to support a positive response to psychological distress [24]. Researches [25–27] showed mindfulness exercise can improve the nurse’ well-being, socialization, anxiety, fatigue, secondary traumatic stress levels and job burnout. But some reviews also showed that the effects of the intervention of self-care and mindfulness depends on the nurses’ psychological inflexibility [28] and physical health status [20]. And providing strategies for self-care and mindfulness at work will be useful if only nurses value and have the opportunity to make use of them [29]. So what’s more important is to identify whether nurses value the self-care strategies and what kinds of strength or weakness they have in the strategies.

To help individuals identify areas of strength and weakness in mindful self-care and to improve self-care strategies, Cook-Cottone and Hotchkiss developed Mindful Self-Care Scale [19, 30]. The concept of Mindful self-care is the integration of mindfulness and traditional self-care practices that involves mindful self-awareness and assessment of one’s internal and external needs. It also include the intentional engagement in specific practices of self-care to address needs and demands in a manner that serves one’s well-being and personal effectiveness [31]. The sub-scales of MSCS fit well with Maslow’s theory and have strong internal consistency reliability [19]. Researches found Mindful Self-Care were the strongest protective factors against burnout and the secondary traumatic stress in Hospice Care Professionals [21] and Chaplains [32]. To improve mental health of oncology nurses using self-care and mindfulness, the very important thing should be explore the situation of their mindful self-care ability. Yang Z [33]. has translated and tested the psychometric properties of the B-MSCS among Chinese hospice nurses. However, the investigation and in-depth analysis of mindful self-care among oncology nurses in China is lacking. LPA is a categorical latent variable modeling approach that focuses on identifying latent sub-populations within a population based on a certain set of variables [34]. Compared to traditional, non-latent clustering methods, LPA can classify individuals into clusters based on membership probabilities estimated directly from the model and demographics, and other covariates can be used for profile description. It has been widely applied in psychology and humanities research to identify types of people who have divergent personal attribute profiles [35]. This research used LPA to better understand the heterogeneity of oncology nurses’ mindful self-care ability and demographic differences related to mindful self-care, which will help us identify areas of strengths and weaknesses in oncology nurses’ mindful self-care abilities and develop improvement strategies.

Accordingly, this study aimed to identify heterogeneity groups of oncology nurses on mindful self-care ability based on latent profile analysis and examine the sociodemographic correlation of these profiles.

## Methods

### Study design

This study was a cross-sectional analysis. This study was designed and reported in accordance with Checklist for Reporting Results of Internet E-Surveys (CHERRIES) [36]. And the checklist was attached as supplementary file 1.

### Settings

The study was conducted in the oncology department of Henan Province, which includes two affiliated comprehensive hospitals and one affiliated oncology hospital.

### Participants

Because the LPA requires a sample size greater than 500 [37], the sample size for this study was at least 500. An online anonymous cross-sectional survey of 911 oncology nurses in two university-affiliated comprehensive hospitals and one university-affiliated cancer hospital was conducted from January to May 2023. All oncology nurses who met the inclusion criteria for the study were invited to take part in the survey. The following criteria have to be met in order to be eligible to take part in this study: (1) a full-time oncology nurses with professional qualifications, (2) have worked in the oncology department for more than 2 years, (3) willing to participate in the study. The exclusion criteria for were as follows : (1) nurses who were rotating to the oncology department, (2) nurses from external hospitals who come for continuous studies.

### Data collection tools

#### Demographic questionnaire (DQ)

The questionnaire included several questions to collect data on participants’ age, sex, education level, marital status, professional title, years of being in the profession, and so on.

#### The brief mindful self-care scale

The B-MSCS is a 24-item scale that measures the self-reported frequency of self-care behaviors using Likert-type scale response anchors (1= “never”; 5= “always”) [30]. This scale was the result of exploratory and confirmatory factor analyses with large samples. The subscales of the B-MSCS include physical care (PC), supportive relationships (SR), mindful self-awareness (MA), self-compassion and purpose (SCP), mindful relaxation (MR), and support structure (SS). The Cronbach’s αs for the subscales were 0.77, 0.77, 0.86, 0.78, 0.74 and 0.79. The B-MSCS total scale and subscale have strong internal consistency and reliability [19]. Yang [33] translated the scale into Chinese and validated its reliability and validity among hospice nurses. The Cronbach’s α value of the Chinese version of B-MSCS was 0.92, and the Cronbach’s α value of the dimensions ranged from 0.85 to 0.93. The split-half reliability and test-retest reliability were 0.77 and 0.72. The content validity index of the scale (S-CVI) was 0.95. The 6-factor structure, supported by the eigenvalues, total variance explained, and scree plot were obtained by using exploratory factor analysis. As a result of the confirmatory factor analysis, the model fitting indexes were all in the acceptable range. Because it was first used in oncology nurses, the scale was first validated.

### Data collection

To recruit study participants, the project leader contacted the chief head nurse of each oncology department and met with them to let them provide feedback on the questionnaire. Wenjuanxing (www.wjx.cn), a popular online data gathering website in China, was utilized to produce a web survey. Questionnaires were distributed to participants using WeChat, a well-known social media platform in China. Before completing the questionnaire, each participant was provided an informed consent about data confidentiality, storage, and the purpose of the study. To avoid duplication, only one questionnaire could be completed per IP address. There was no time limit for completing the questionnaire. All items in the questionnaire were set as mandatory in Wenjuanxing, so participants must answer all questions before submitting the questionnaire. After completing the questionnaire, the participants got the rewards which were set by researcher through the website. The questionnaires that takes less than 70s to complete were considered invalid according to a cut score for response time at 2 s an item [38]. The response times too slow were not been considered because many more factors may contribute to this situation [39]. Because the item 4 of the B-MSCS is a reverse question, the questionnaires which does not conform to item 2 were also regarded as invalid. According to the principle of confidentiality, only researchers have the account number and password to enter the website to see the data. A final total of 839 valid questionnaires were returned, resulting in an effective response rate of 92.10%.

### Data analysis

The statistical analysis was performed using AMOS 21, Mplus 7.4, and IBM SPSS Statistics 25.0. Descriptive statistics were conducted to summarize participants’ demographic, clinical characteristics and scores on the scales, including the numbers, frequencies, means and standard deviations(SDs). AMOS 21 was used to conduct confirmatory factor analysis(CFA) to examine whether the construct of the instrument tested among Chinese oncology nurses would be in accordance with the original scale.

The results of the 24 items on the B-MSCS served as exogenous variables. Because the B-MSCS scores were continuous variables, latent profile analysis (LPA) was carried out using the Mplus 7.4 program. The following fit indicators were used to determine the optimal number of latent profiles: Log Likelihood (LL), Akaike Information Criterion (AIC), Bayesian Information Criterion (BIC) and adjusted Bayesian Information Criterion (aBIC) were used to compare models, with lower AIC, BIC, and aBIC values indicating a better model fit. The Lo-Mendell-Rubin (LMR) and Bootstrapped Likelihood Ratio Test (BLRT) were used to determine whether a k-class model fit better than a model with k-1 classes, and a significant p value indicated that the k class was better. The entropy values were assessed to determine the classification precision of each model, with entropy values greater than 0.80 indicating adequate classification precision. The minimum percentage of potential subgroups should not be less than 5%. The fitting results of each class and the needs of the researchers were considered together to determine which was the best model.

The SPSS 25.0 statistical program was used to conduct One-way ANOVA and multinomial logistic regression. Frequency and composition ratios were used to describe the categorical variables. The mean and standard deviation were used to describe the continuous variables. One-way ANOVA was used to compare the scores on each B-MSCS dimension in each profile. When significant between-group effects were observed, and post hoc analyses were performed using the Tukey HSD method. One-way ANOVA and the chi-square test were performed to compare differences in sociodemographic characteristics among the subgroups. Multinomial logistic regression was subsequently performed to examine the influence of the sociodemographic variables on each profile. A p value < 0.05 indicated statistical significance for all analyses.

### Ethical considerations

The study was conducted in accordance with the Declaration of Helsinki. Informed consent was obtained from all participants before their enrollment in the study. The study received the approval of the Ethics Review Committee of the School of Nursing and Health, Zhengzhou University (approval number 2022 − 131).

## Results

### Participants’ characteristics

Most of the respondents were female (98.8%) and aged less than 40 (94.8%). A total of 91.4% of them had no religious beliefs. Over half of the respondents (68.9%) were married and had a bachelor’s degree (53.8%). Table 1 shows the demographic profiles of the participants.

**Table 1:** Demographic profiles of the participants (*n* = 839)

Variables	Mean(SD)	*n*(%)
**Gender**		
Male		10(1.2)
Female		829(98.8)
**Age**	31.24(5.54)	
**Marital status**		
Married		578(68.9)
Unmarried		252(30.0)
Separated/divorced		9(1.1)
**Education Level**		
Bachelor degree		451(53.8)
Master degree		22(2.6)
Doctor degree		1(0.1)
Without degree		365(43.5)
**Professional title**		
Nurse		131(15.6)
Nurse-in-charge		688(82.0)
Co-Chief Nurse		18(2.2)
Chief Nurse		2(0.2)
**Position**		
Nurse		762(90.8)
Head nurse		54(6.4)
Deputy Director of Nursing		4(0.5)
Director of Nursing		3(0.4)
Others		16(1.9)
**Years of being in the profession**		
2–5		191(22.8)
6–10		354(42.2)
11–15		294(35.0)
**Income per month(CNY)**		
1000–3000		47(5.6)
3001–5000		64(7.6)
5001–7000		134(16.0)
7001–9000		323(38.5)
Above 9000		271(32.3)
**Be concerned about self-care**		
Yes		503(60.0)
No		336(40.0)
**Be interested in mindfulness**		
Yes		455(47.7)
No		384(52.3)
**Having experience of Meditation**		
Yes		400(47.7)
No		439(52.3)

### The validation results of the Chinese version of B-MSCS among oncology nurses

The Cronbach’s α of the whole scale in this research was 0.93, and the index of the six sub-scales ranged from 0.70 to 0.94. Table 2 presents the results of testing the reliability of the Chinese version of B-MSCS among oncology nurses.

**Table 2:** Internal consistency of the Chinese version of B-MSCS in this research

Variables	Number of items	Score of B-MSCS in this research (*N* = 839)	Cronbach’s alpha of each dimension (*N* = 839)
Physical Care (PC)	5	12.86 ± 3.30	0.70
Supportive Relationships (SR)	4	13.22 ± 3.09	0.83
Mindful Self-Awareness (MS)	3	10.54 ± 2.36	0.94
Self-Compassion and Purpose (SCP)	4	13.63 ± 2.87	0.88
Mindful Relaxation (MR)	4	11.73 ± 3.15	0.82
Support Structure (SS)	4	14.41 ± 2.75	0.87

The data was also validated using confirmatory factor analysis(CFA), the model obtained a good fit. The χ^2^/df was 2.022, which is less than the strict index value of three (*P* < 0.001). The RMSEA was 0.049, which is less than 0.05. Other indices were: GFI = 0.915, TLI = 0.955, AGFI = 0.890, CFI = 0.963, and NFI = 0.929. Only AGFI was lower than 0.9, but it was close to 0.9.

### The dimensions of the level of mindful self-care of oncology nurses

The total score of the B-MSCS was 76.40 ± 13.19. The SS dimension had the highest score, with an average mean value of 3.60, followed by the MA, SCP, SR, MR and PC dimensions. PC had the lowest score at 2.57. Table 3 shows the details.

**Table 3:** The level of mindful self-care of oncology nurses(*N* = 839)

Variables	Rank	Number of the items	Average Score of item of B-MSCS in oncology nurses (x ± S)
Support structure(SS)	1st	4	3.60 ± 0.69
Mindfulness self-awareness(MA)	2nd	3	3.51 ± 0.79
Self-compassion and purpose(SCP)	3rd	4	3.41 ± 0.72
Supportive relationship(SR)	4th	4	3.31 ± 0.77
Mindful relaxation(MR)	5th	4	2.93 ± 0.79
Physical care(PC)	6th	5	2.57 ± 0.66

### Characteristics of the different profiles

**Fig. 1:**
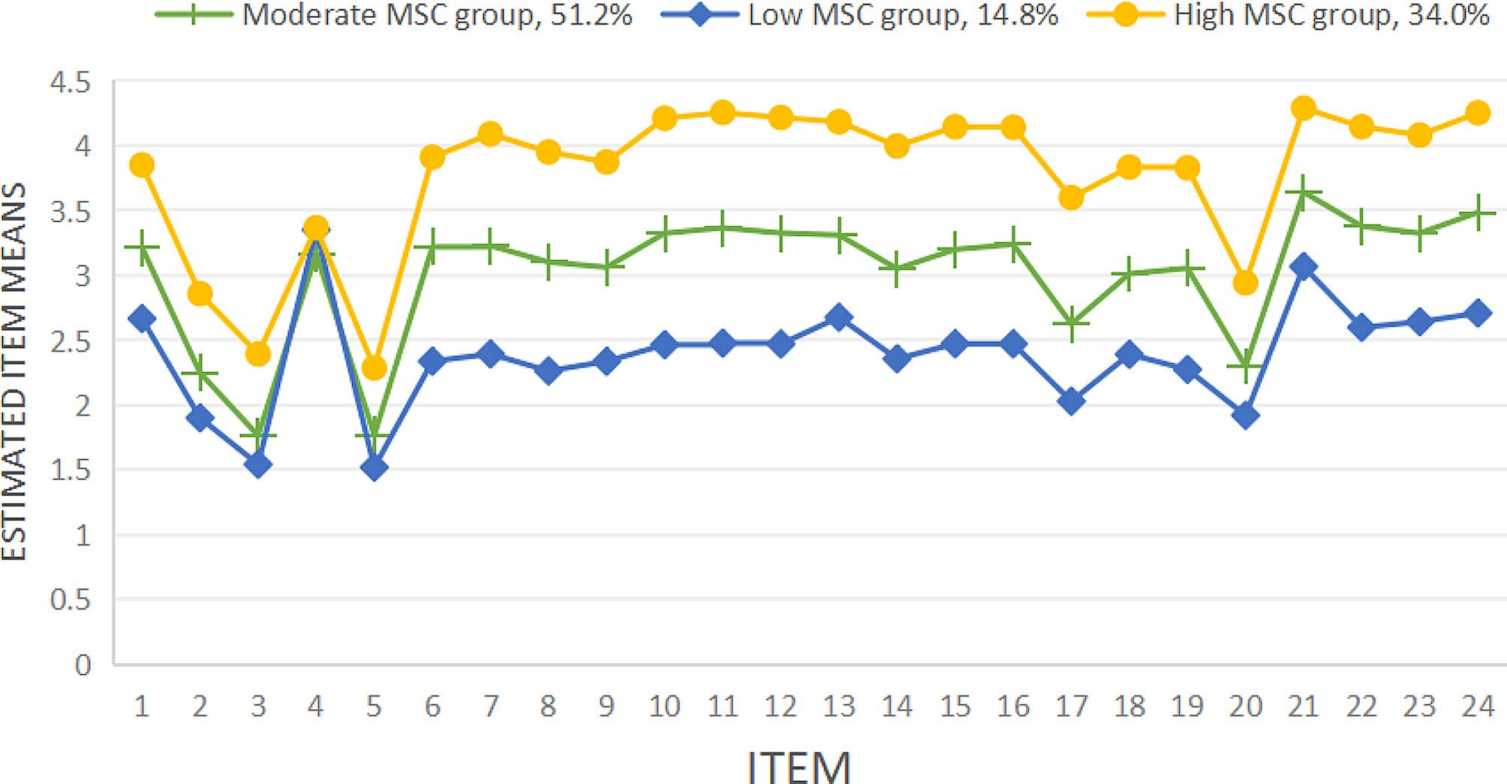
Latent profiles of MSC

Table 4 shows the model fit indices for the one-class to four-class solutions. The LL, AIC, BIC, and aBIC generally decreased as the number of estimated profiles increased, while the entropy remained above 0.90 consistently. The LMR tests were significant except for Model 4, which showed that the Model 4 solution did not enhance the model fit considerably compared with that of the Model 3 solution (*p* = 0.1142). The BLRT results were all significant. Model 3, which included three latent categories(C1, C2, and C3), was determined to be the model with the best fit indices when the models’ fit indices were evaluated. Based on the results of the latent profile analysis, the scores of the three profiles on the 24 items of the B-MSCS were plotted, and their characteristics are summarized in Fig. 1. C1, C2, and C3 were designated in accordance with their distinctive distributions based on the mean scores of the B-MSCS in each profile. C1 accounted for approximately 51.2% of the total number of subjects, and its score on each item was between C2 and C3. Therefore, this category was named “moderate mindful self-care” based on its score characteristics. C2 accounted for approximately 14.8% of the total, and its score on each item was lower than C1 and C3 and was therefore named “low-low Mindful relaxation” based on its score characteristics. C3 accounted for 34.0% of the total, and its score was significantly higher than that of C1 and C2. This category was therefore named “high-high Mindfulness self-awareness” based on its score characteristics.

The latent profile memberships exhibited significant differences in the means of the six indicator variables (*p* < 0.001). A post hoc analysis using the Tukey HSD method revealed that oncology nurses in the C3 group scored higher than those in the C1 and C2 group on both the total scale score and each dimension. The C1 group also scored higher than the C2 group. Table 5 showed the details.

**Table 4:** Potential profile analysis indicators (*N* = 839)

Model	LL^a^	AIC^b^	BIC^c^	aBIC^d^	Entropy	LMR^e^ *p* value	BLRT^f^ *p* value	Category probability (%)
Model 1	-26420.678	52937.356	53164.502	53012.070				
Model 2	-23806.502	47759.004	48104.456	47872.632	0.929	<0.001	<0.001	57.2/42.8
**Model 3**	**-23078.718**	**46353.436**	**46817.193**	**46505.977**	**0.930**	**<0.001**	**<0.001**	**51.2/14.8/34.0**
Model 4	-22585.191	45416.382	45998.444	45607.836	0.935	0.1142	<0.001	9.6/38.1/42.3/10.0

Note.^a^ Log likelihood, ^b^ Akaike information criterion, ^c^ Bayesian information criterion, ^d^ Adjusted Bayesian information criterion, ^e^ Lo-Mendell-Rubin likelihood ratio test, ^f^ Bootstrapped likelihood ratio test. The boldface text indicates the selected model

**Table 5:** B-MSCS scores and dimensions in different categories (*N* = 839)

Variables	Class1 Mean(SD)	Class2 Mean(SD)	Class3 Mean(SD)	F	*p*
Physical care(PC)	12.16(2.58)	10.98(2.55)	14.77(3.63)	95.331	<0.001
Supportive relationship(SR)	12.62(2.09)	9.27(2.27)	15.85(2.26)	428.447	<0.001
Mindfulness self-awareness(MA)	10.01(1.53)	7.44(1.64)	12.68(1.58)	539.343	<0.001
Self-compassion and purpose(SC)	12.90(1.66)	9.98(2.08)	16.48(1.81)	673.466	<0.001
Mindful relaxation(MR)	11.01(2.27)	8.54(2.33)	14.21(2.78)	267.002	<0.001
Support structure(SS)	13.83(1.90)	11.01(2.31)	16.78(1.84)	416.494	<0.001
Total score of B-MSCS	72.43(5.26)	57.21(6.90)	90.76(7.72)	1341.827	<0.001

*Notes.*Class 1: Moderate Mindful Self-Care, Class 2: Low-Low Mindful Relaxation,

Class 3: high-high mindfulness self-awareness

### Demographic and related characteristics of each profile

Table 6 showed the results of the univariate analysis which revealed that significant differences between the three categories regarding professional title(Χ^2^= 14.820, *p* = 0.022), position(Χ^2^= 19.990, *p* = 0.010), whether concerned about self-care(Χ^2^= 30.430, *p*<0.001), whether interested in mindfulness(Χ^2^= 32.691, *p*<0.001), and experience of meditation(Χ^2^= 23.814, *p*<0.001).

A multinomial logistic regression analysis was carried out using C3 group as the reference group to pinpoint the variables connected to MSC among the three categories. The professional title and position were converted to binary to avoid floating-point overflow. The outcomes are displayed in Table 7.

The findings showed that when compared to those who were not concerned about self-care, oncology nurses concerned about self-care had lower odds of being in the C1 group and C2 group than in the C3 group (OR: 0.643, CI: 0.446–0.928 and OR: 0.558, CI: 0.340–0.916, *p*<0.05). Compared to those who were not interested in mindfulness, oncology nurses interested in mindfulness had lower odds of being in the C1 group and C2 group (OR: 0.690, CI: 0.480–0.993 and OR: 0.536, CI: 0.324–0.888, *p*<0.05). Compared to those who were not experienced with meditation, oncology nurses with meditation experience had lower odds of being in the C1 group and C2 group than in the C3 group (OR: 0.715, CI: 0.515–0.993, *p*<0.05; OR: 0.521, CI: 0.325–0.834, *p*<0.01).

**Table 6:** Demographics and characteristics by latent profile (*N* = 839)

Variables	Class 1	Class 2	Class 3	Χ^2^/F	*p*
**Gender**				0.354	0.838
Female	424(98.6%)	123(99.2%)	282(98.9%)		
Male	6(1.4%)	1(0.8%)	3(1.1)		
**Age**	30.98(5.19)	30.77(4.00)	31.83(6.53)	2.510	0.082
**Marital status**				0.985	0.912
Married	297(69.1%)	86(69.4%)	195(68.4%)		
Unmarried	127(29.5%)	37(29.8%)	88(30.9%)		
Separated/divorced	6(1.4%)	1(0.8%)	2(0.7%)		
**Education Level**				8.347	0.214
Bachelor degree	230(53.5%)	63(50.8%)	158(55.4%)		
Master degree	10(2.3%)	2(1.6%)	10(3.5%)		
Doctor degree	0(0)	1(0.8%)	0(0)		
Without degree	190(44.2%)	58(46.8%)	117(41.1%)		
**Professional title**				14.820	0.022
Nurse	77(17.9%)	17(13.7%)	37(13.0%)		
Nurse-in-charge	345(80.2%)	107(86.3%)	236(82.8%)		
Co-Chief Nurse	6(1.4%)	0(0)	12(4.2%)		
Chief Nurse	2(0.5%)	0(0)	0(0)		
**Position**				19.990	0.010
Nurse	398(92.6%)	120(96.8%)	244(85.6%)		
Head nurse	20(4.7%)	3(2.4%)	31(10.9%)		
Deputy Director of Nursing	2(0.5%)	1(0.8%)	1(0.4%)		
Director of Nursing	2(0.5%)	0(0)	1(0.4%)		
Others	8(1.9%)	0(0)	8(2.8%)		
**Years of being in the profession**				7.558	0.109
2–5	105(24.4%)	21(16.9%)	65(22.8%)		
6–10	180(41.9%)	64(51.6%)	110(38.6%)		
11–15	145(33.7%)	39(31.5%)	110(38.6%)		
**Income per month(CNY)**				4.234	0.835
1000–3000	26(6.0%)	6(4.8%)	15(5.3%)		
3001–5000	32(7.4%)	9(7.3%)	23(8.1%)		
5001–7000	73(17.0%)	17(13.7%)	44(15.4%)		
7001–9000	164(38.1%)	56(45.2%)	103(36.1%)		
Above 9000	135(31.4%)	36(29.0%)	100(35.1%)		
**Concern about self-care**				30.432	<0.001
Yes	239(55.6%)	58(46.8%)	206(72.3%)		
No	191(44.4%)	66(53.2%)	79(27.7%)		
**Interest in mindfulness**				32.691	<0.001
Yes	215(50%)	49(39.5%)	191(67.0%)		
No	215(50%)	75(60.5%)	94(33.0%)		
**Experience with meditation**				23.814	<0.001
Yes	192(44.7%)	42(33.9%)	166(58.2%)		
No	238(55.3%)	82(66.1%)	119(41.8%)		

*Notes.*Class 1: Moderate Mindful Self-Care, Class 2: Low-Low Mindful Relaxation,

Class 3: high-high mindfulness self-awareness

**Table 7:** Multinomial logistic regression analysis of potential categories of oncology nurses’ mindful self-care(*N* = 839)

Variables	Class1 vs. Class3			Class2 vs. Class3		
	β	OR	95%CI	β	OR	95%CI
**Age**	0.009	1.010	0.975–1.045	-0.005	0.995	0.945–1.048
**Professional title**						
Nurse	0.374	1.454	0.891–2.371	-0.045	0.956	0.472–1.934
Nurse-in-charge and above						
**Position**						
Nurse	0.648	1.912	0.991–3.668	0.920	2.508	0.780–8.064
Head nurse and above						
**Concern about self-care**						
Yes	-0.441	0.643*	0.446–0.928	-0.584	0.558*	0.340–0.916
No						
**Interest in mindfulness**						
Yes	-0.371	0.690*	0.480–0.993	-0.623	0.536*	0.324–0.888
No						
**Experience with meditation**						
Yes	-0.335	0.715*	0.515–0.993	-0.652	0.521**	0.325–0.834
No						

*p<0.05 **p<0.01

Note. Class 1: Moderate Mindful Self-Care, Class 2: Low-Low Mindful Relaxation,

Class 3: high-high mindfulness self-awareness

OR, odds ratio; 95% CI, 95% confidence interval; ref, reference

## Discussion

As far as we know, this is the first study to look into the co-occurrence of different types of mindful self-care among oncology nurse. We used the LPA technique to group oncology nurses by their level of mindful self-care ability and identified three distinct profiles—moderate mindful self-care(C1), low-low Mindful relaxation(C2), and high-high Mindfulness self-awareness(C3). Each group’s average score 72.43, 57.21, and 90.76.

The group with the highest proportion was the moderate mindful self-care group which was Class 1. In this group, the score of Physical care(PC) dimension was the lowest indicating the physical care was a weakness for half of oncology nurses. In the concept of mindful self-care, PC emphasizes on basic nutrition, hydration, and exercise practices [30], which are related to people’s mood and reduce stress [40]. And as researches showed, practices like yoga can integrates bottom-up neurophysiological and top-down neurocognitive mechanisms helping individuals achieve self-regulation and resilience [41]. On the contrary, physical inactivity leads to a range of adverse health consequences [42]. Nursing manager can encourage oncology nurses to take part in physical activities, regular exercise, sports, mind-body practice and other activities. On the other hand, in this moderate mindful self-care group, the score of support structure(SS) dimension was the highest. The SS dimension emphasizes environmental factors, which include keeping work areas organized, constructing a manageable schedule and maintaining a pleasing and comfortable living environment [30]. This can be explained by the professional competence requirements of nurses, and also suggested that half of the oncology nurses have more conscious of balancing work and life. These features should be fully utilized in the future intervention.

14.8% of oncology nurses were in the low-low Mindful relaxation group which was Class 2. We found that the Mindful Relaxation(MR) dimension was lower in the C2 group than other dimensions of the group. MR can be described as a technique for self-soothing, calming, and relaxation, which are believed to be effective tools in emotional regulation [30]. And it has been linked negatively to sleeping problems, and emotional exhaustion, but positively to work performance and job satisfaction [43]. MR includes doing something creative or intellectual, listening, and soughing out images or smells to relax. The creative or intellectual activities include doing art activities, playing musical instruments, doing creative writing, singing or cleaning. Researches showed that music intervention and playing music can be a cost-effective resource for reducing stress and improving the well-being of healthy people and oncology nurses [44–46]. Storytelling through music can also address work-related emotions and psycho-social stress in oncology nurses [47]. Additionally, mindful cleaning, such as organizing drawers, washing dishes, or mopping floors, can allow people to experience greater state mindfulness [48], increase people’s mood and reduce their anxiety [49]. Smells, including using essential oils, being exposed to nature, lighting candles, and baking, can affect mood and psychiatric disorders, such as depression and anxiety, and can improve people’s connection to nature and well-being [50]. And the research on Zentangle art intervention [25] also showed its effects on the improvement of oncology nurses’ well-being, socialization, anxiety, fatigue, and secondary traumatic stress levels. In the future, mindfulness self-care interventions for oncology nurses in the low-low Mindful relaxation group could focus on these aspects. In terms of items, the score of item 4 ‘I did sedentary activities instead of exercising’, which was a reverse question, got the highest score compared to other groups. This indicated that oncology nurses, even through in this low MSC group, never thought they were sedentary and the concept ‘exercise’ in their mind may include walking and running in the workplace, which oncology nurses do every day. The item 2, ‘I exercised at least 30 to 60 min’ was also not very low. These suggests the concept of Physical care(PC) needs to be emphasized and clarified in future interventions. Compared with other groups, the item 13 ‘I calmly accepted the challenges and difficulties in my life’ was higher. In other words, although mindful self-care ability was low, oncology nurses in this group have more potential in maintaining work environment, completing tasks and facing challenges and difficulties.

34.0% of oncology nurses were classified as the high-high Mindfulness self-awareness group which was Class 3. We found that C3 group scored higher on the dimensions of mindfulness self-awareness(MA) compared to others dimensions. Mindful self-awareness was described as fundamental and unique features of mindful self-care [30, 51] which happens to explain why the MA dimension got the highest score in this high MSC group. In the mindful self-care scale, mindful self-awareness dimension assess a calm awareness of thoughts, feelings, and the physical body as well as the careful and intentional selection of thoughts and feelings an individual uses to guide his or her actions [30]. Such abilities can be vehicles for compassion and let oncology nurses have mind full of kindness [52]which help them to recognize patient distress [12]and improve the provision of empathy-based care [53]. In turn, these also can help them have more care for themselves and enhance their positive aspects of psychology. So mindful self-awareness will be the most important aspect in mindfulness self-care interventions for oncology nurses.

Our research also revealed that the three classes differed in terms of their social characteristics, including professional title and position. But these characteristics were not the influential factors for mindful self-care in oncology nurses according to the results of the multinomial logistic regression analysis. Whether oncology nurses concern about self-care, interest in mindfulness and have experience with meditation were meaningful when C1 and C2 group were compared with C3 group. This finding is consistent with previous research which showed it was essential to improve the knowledge of mindfulness and self-care for health care professionals [54]. Although mindfulness and self-care are universal psychological abilities of humans, not everyone thinks they are important for life. Only Oncology nurses who value strategies for self-care [32] and have interests will have the motivation to learn. Nursing manager should find interventions to improve the knowledge and strategies of mindful self-care among oncology nurses. Meditation is a kind of mindful practice. And when studied about meditation in the light of neuroscience, our brain indeed has a system through which we can get rid of maladaptive thoughts and restructure our brain was labeled [55]. Researches also indicated meditation is effective at decreasing stress and burnout in nurses [56]. But as other research showed, although meditation and other self-care are clearly beneficial to nurses, but they cannot take the place of organizational support and healthy work [57]. Since experience meditation can influence mindful self-care ability classification of oncology nurses, nursing manager should give opportunities for them to practice meditation.

### Limitations

This research has several limitations. First, our cross-sectional study utilized only convenience sampling, and the questionnaire was self-reported and web-based. This may cause bias. Random sampling should be used in further research. Second, in addition to personal characteristics, this research did not use other scales to determine the potential influencing factors of mindful self-care. Based on relative theory, further research should be performed to determine the relationships among other factors and to explore the mesomeric or moderating effects of these factors. Research on interventions for mindful self-care among oncology nurses can also be performed further in China.

## Conclusions

This study used latent profile analysis to identify the category characteristics and the influencing factors of oncology nurses’ mindful self-care ability. The mindful self-care among oncology nurses can be categorized into three latent profiles: moderate mindful self-care(51.2%), low-low mindful relaxation(14.8%), and high-high mindfulness self-awareness(34.0%). Multinomial logistic regression results indicated that whether oncology nurses concern about self-care, interest in mindfulness and have experience with meditation influenced different latent profiles. These findings provide new insights in the mindful self-care among oncology nurses and may help nursing managers to implement some new management policies or mindful based intervention.

### Electronic supplementary material

Below is the link to the electronic supplementary material.

Supplementary Material 1

## Data Availability

The data supporting the findings of this study are available on request from the corresponding author. The data are not publicly available due to privacy or ethical restrictions.
